# Supplementary material for: Werner syndrome helicase is a selective vulnerability of microsatellite instability-high tumor cells
Source: eLife. 2019 Mar 25;8:e43333. doi: 10.7554/eLife.43333 (PMC6435321; doi:10.7554/eLife.43333)
Supplement: Supplementary file 1. — MSS/MSI-H status was analyzed using fluorescent PCR-based analysis of the mononucleotide microsatellite markers NR-21, BAT-26, BAT-25, NR-24 and MONO-27. Main peak sizes for the mononucleotide microsatellite markers are shown for the MSS control cell line K562 and CRC, endometrial and gastric carcinoma cell lines. Cell models were classified as MSS (blue) or MSI-H (red) according to the indicated size range classification of MSS alleles. [file elife-43333-supp1.docx]

**Supplementary File 1**

|  |  | **MSS control cell line** | **Normal retinal pigment epithelial cells** |  |  |  |
| --- | --- | --- | --- | --- | --- | --- |
| **Marker** | Size range (bp) | K562 (bp) | hTERT RPE-1 (bp) |  |  |  |
| **NR-21** | 94-101 | 100.6 | 100.7 |  |  |  |
| **BAT-26** | 103-115 | 113.4 | 114.4 |  |  |  |
| **BAT-25** | 114-124 | 121.5 | 121.6 |  |  |  |
| **NR-24** | 130-133 | 130.1 | 130.0 |  |  |  |
| **MONO-27** | 142-154 | 149.9 | 149.0 |  |  |  |
|  |  |  |  |  |  |  |
|  | **CRC cell lines** | | | |  |  |
| **Marker** | SK-CO-1  (bp) | SW480  (bp) | CaCo-2 (bp) | HCT 116  (bp) | RKO  (bp) | SNU-C4 (bp) |
| **NR-21** | 100.6 | 99.6 | 99.7 | 92.3 | 87.0 | 90.3 |
| **BAT-26** | 114.4 | 113.4 | 113.5 | 101.9 | 102.7 | 101.8 |
| **BAT-25** | 121.5 | 120.5 | 121.8 | 115.2 | 112.1 | 114.2 |
| **NR-24** | 130.1 | 130.1 | 131.1 | 120.9 | 124.0 | 120.8 |
| **MONO-27** | 148.9 | 149.9 | 149.1 | 140.7 | 137.6 | 138.7 |
|  |  |  |  |  |  |  |
|  | **Endometrial carcinoma cell lines** | | | |  |  |
| **Marker** | MFE-280  (bp) | HEC-265  (bp) | ISHIKAWA  (bp) | HEC-6 (bp) |  |  |
| **NR-21** | 99.6 | 91.3 | 88.1 | 89.1 |  |  |
| **BAT-26** | 113.4 | 102.0 | 100.9 | 100.8 |  |  |
| **BAT-25** | 122.6 | 117.4 | 113.1 | 112.0 |  |  |
| **NR-24** | 131.1 | 123.9 | 123.0 | 123.0 |  |  |
| **MONO-27** | 148.9 | 143.8 | 140.7 | 141.7 |  |  |
|  |  |  |  |  |  |  |
|  | **Gastric carcinoma cell lines** | | | |  |  |
| **Marker** | AGS (bp) | OCUM-1 (bp) | 23132.87 (bp) | IM95 (bp) |  |  |
| **NR-21** | 98.6 | 99.6 | 89.2 | 94.4 |  |  |
| **BAT-26** | 113.4 | 113.4 | 101.9 | 101.9 |  |  |
| **BAT-25** | 121.5 | 121.6 | 114.2 | 114.2 |  |  |
| **NR-24** | 131.2 | 131.1 | 123.0 | 119.8 |  |  |
| **MONO-27** | 149.9 | 150.0 | 143.7 | 140.7 |  |  |
