# Supplementary material for: Werner syndrome helicase is a selective vulnerability of microsatellite instability-high tumor cells
Source: eLife. 2019 Mar 25;8:e43333. doi: 10.7554/eLife.43333 (PMC6435321; doi:10.7554/eLife.43333)
Supplement: Supplementary file 2. — Cell lines used in this study are listed with tumor type of origin, MSS/MSI-H status, vendor source, and STR confirmation status. Variable STR profiles are reported for ISHIKAWA cells, consistent with MSI-H status (Korch et al., 2012). [file elife-43333-supp2.docx]

**Supplementary File 2**

| **Cell line** | **Tumor type** | **Source** | **STR Confirmed** | **MSI/MSS status** | **Reference for MSI/MSS status** |
| --- | --- | --- | --- | --- | --- |
| CaCo-2 | CRC | ATCC | Yes | MSS | Medico et al. (2015) Nat Commun 6: 7002, this study |
| HCT 116 | CRC | ATCC | Yes | MSI-H | Medico et al. (2015) Nat Commun 6: 7002, this study |
| HCT 116 _CRISPR-Cas9-Puro | CRC | This study | Yes | MSI-H | Medico et al. (2015) Nat Commun 6: 7002, this study |
| HEC-265 | Endometrial carcinoma | JCRB 1142 | Yes | MSI-H | This study |
| HEC-6 | Endometrial carcinoma | JCRB 1118 | Yes | MSI-H | This study |
| HT-29_CRISPR-Cas9-Blasti | CRC | This study | Yes | MSS | Medico et al. (2015) Nat Commun 6: 7002 (parental line) |
| hTERT RPE-1 | Normal retinal pigment epithelial cells | ATCC | Yes | MSS | This study |
| ISHIKAWA | Endometrial carcinoma | ECACC | No* | MSI-H | This study |
| LS1034 | CRC | ATCC | Yes | MSS | Medico et al. (2015) Nat Commun 6: 7002 |
| MFE-280 | Endometrial carcinoma | DSMZ | Near full | MSS | This study |
| RKO | CRC | ATCC | Yes | MSI-H | Medico et al. (2015) Nat Commun 6: 7002, this study |
| RKO Cas9-puro | CRC | ATCC | Near full | MSI-H | Medico et al. (2015) Nat Commun 6: 7002, this study (parental line) |
| SK-CO-1 | CRC | ATCC | Yes | MSS | Medico et al. (2015) Nat Commun 6: 7002, this study |
| SK-CO-1 Cas9-puro | CRC | ATCC | Yes | MSS | Medico et al. (2015) Nat Commun 6: 7002, this study |
| SNU-C4 | CRC | KCLRF | Yes | MSI-H | Medico et al. (2015) Nat Commun 6: 7002, this study |
| SW480 | CRC | ATCC | Near full | MSS | Medico et al. (2015) Nat Commun 6: 7002, this study |
| SW480 wild-type (monoclonal) | CRC | This study | N/A | MSS | Medico et al. (2015) Nat Commun 6: 7002 (parental line) |
| SW480 WRN KO #1 | CRC | This study | N/A | MSS | Medico et al. (2015) Nat Commun 6: 7002, this study (parental line) |
| SW480 WRN KO #2 | CRC | This study | N/A | MSS | Medico et al. (2015) Nat Commun 6: 7002, this study (parental line) |
| HCT 116 +ch2 | CRC | Koi et al. (1994) Cancer Res 54(16): 4308-4312. | N/A | MSI | Medico et al. (2015) Nat Commun 6: 7002, this study (parental line) |
| HCT 116 +ch3 | CRC | Koi et al. (1994) Cancer Res 54(16): 4308-4312. | N/A | N/A (MLH1 reconstitution) | Medico et al. (2015) Nat Commun 6: 7002, this study (parental line) |
| HCT 116 +ch3 +ch5 | CRC | Haugen et al. (2008) Cancer Res 68(20): 8465-8472. | N/A | N/A (MLH1/MSH3 reconstitution) | Medico et al. (2015) Nat Commun 6: 7002, this study (parental line) |

* variable STR profiles reported consistent with MSI-H status (Korch et al. (2012) Gynecol Oncol 27(1):241-8)
