# Supplementary material for: Werner syndrome helicase is a selective vulnerability of microsatellite instability-high tumor cells
Source: eLife. 2019 Mar 25;8:e43333. doi: 10.7554/eLife.43333 (PMC6435321; doi:10.7554/eLife.43333)
Supplement: Supplementary file 3. — Sequences of sgRNAs used for targeting WRN are listed in N- to C-terminal order according to the representation in Figure 3 and Expanded View Figure 3. Domains are annotated according to PFAM entry Q14191. RQC, RecQ helicase family DNA-binding domain; HRDC, Helicase and RNase D C-terminal, HTH, helix-turn-helix motif. Negative and positive control sgRNA sequences are also listed. [file elife-43333-supp3.docx]

**Supplementary File 3**

| **WRN domain targeting sgRNAs (N- to C-terminal order)** | |
| --- | --- |
| **Targeted domain** | **sgRNA sequence** |
| **Exonuclease** | AGTCTATCCGCTGTAGCAAT |
|  | GACCTGGAGCCTTAACAGTC |
|  | AACCAGACTGTTAAGGCTCC |
|  | AGTCTGGTTAAACACCTCTT |
|  | GGCCACCATTATACAATAGA |
| **Helicase** | GCTCACTGTATTTCTGAGTG |
|  | AGGCTCACTGTATTTCTGAG |
|  | CTCACTGTATTTCTGAGTGG |
|  | ATGATTTTAGGGATTCATTC |
|  | TTTCTGACTGTGCTGATCCA |
|  | CATTCATTACGGTGCTCCTA |
|  | TTACGGTGCTCCTAAGGACA |
|  | TGCTAAAACTCATGCCCGCA |
|  | GCCCGCATGGTATGTTCCAC |
|  | AAGTTCTTGTCACGTCCTCT |
|  | CATTACGTATCTCAGTAAGA |
|  | AGTTCCCATAATTCCCAAGG |
|  | AAAGCCTCCTTGGGAATTAT |
|  | ACAAGTACAAAAAGCCTCCT |
|  | ATCTTCATTCTAGCAGATGT |
| **RQC** | GTCTTGCCGATCAATATCGC |
|  | ACTGTGCCTGCGATATTGAT |
|  | GACATCTTAGGCGAAAAATT |
|  | AGGCACAGTTTATTTGGCAC |
|  | TATCGCAGGCACAGTTTATT |
| **HRDC** | CTACGGTTGAAAACGTAAAA |
|  | TTTACGTTTTCAACCGTAGT |
|  | GTTTGTTGCCAGAATAGCTG |
|  | TCTGGCAACAAACAAGATAC |
|  | TTTGTTGCCAGAATAGCTGG |
| **HTH** | CCCCTTGATTTGGAGCGAGC |
|  | TTATCCCAAGCGGTGAAAGC |
|  | GGCAGCCAGCTTTCACCGCT |
|  | GGCCTGCTCGCTCCAAATCA |
|  | CTGCTCGCTCCAAATCAAGG |
| **Control sgRNAs** | **sgRNA sequence** |
| Neg#1 | GGCAGTCGTTCGGTTGATAT |
| Neg#2 | GATACACGAAGCATCACTAG |
| RPA3 | GATGAATTGAGCTAGCATGC |
| PCNA | GGACTCGTCCCACGTCTCTT |
| POLR2A | GTACAATGCAGACTTTGACG |
